# Predictors of Sleep Among Spousal Care Dyads Living With Chronic Conditions

**DOI:** 10.1093/geroni/igab046.3520

**Published:** 2021-12-17

**Authors:** Florence Johnson, Sheria Robinson-Lane, Lianlian Lei, Yin Liu, Yeonsu Song, Seung-won Emily Choi, Toni Antonucci

**Affiliations:** 1 University Of Michigan School of Nursing, Ann Arbor, Michigan, United States; 2 University of Michigan School of Nursing, Ann Arbor, Michigan, United States; 3 University of Michigan, Ann Arbor, Michigan, United States; 4 Utah State University, Logan, Utah, United States; 5 UCLA, Los Angeles, California, United States; 6 Texas Tech University, Lubbock, Texas, United States

## Abstract

Sleep health relates closely to physical health and well-being among older adults with chronic health conditions. However, little is known about the dyadic sleep patterns of these individuals and their spousal caregivers. Secondary analyses of the 2015 National Health and Aging Trends Study and National Study of Caregiving (N= 62 care dyads, mean age 78.59 years for care recipients and 75.77 years for caregivers) were completed to examine the sleep patterns, and related factors, of spousal dyads at both the individual and dyadic levels. Sleep measures included frequency in trouble falling back asleep and insomnia symptoms. Predictors included demographics, depressive symptoms, and positive affect for dyads and contextual factors such as dementia caregiving, care burden and support, neighborhood cohesion, and relationship quality. Multilevel dyadic and actor-partner interdependence models were used to complete analyses. Though intraclass correlation was poor (dementia care dyads ICC=0.123, non-dementia care dyads ICC=0.043), persons with dementia/spousal caregiver dyads (n=102) had more similar sleep and insomnia patterns than dyads with other chronic conditions. Poor sleep among dyads was correlated with higher care burden (β = -0.31, p <.0001), however, better relationship quality marginally enhanced the association (β = -0.23, p = .08). Individual depressive symptoms negatively affected dyadic sleep patterns. However, positive affect only had an actor effect and was related to better individual sleep. Other contextual factors did not affect sleep patterns. These findings suggest the importance of both caregiver and care recipient characteristics on sleep at dyadic levels, particularly those with dementia.

